# Significant abnormal glycemic variability increased the risk for arrhythmias in elderly type 2 diabetic patients

**DOI:** 10.1186/s12902-021-00753-2

**Published:** 2021-04-27

**Authors:** Jianbo Zhang, Jianmin Yang, Liwei Liu, Liyan Li, Jiangyin Cui, Shuo Wu, Kuanxiao Tang

**Affiliations:** 1 Department of General Medicine, Qilu Hospital of Shandong University, No.107, Wen Hua Xi Road, Jinan, Shandong China; 2 Department of Emergency and Chest Pain Center, Qilu Hospital, Cheeloo College of Medicine, Shandong University, Jinan, Shandong China; 3 The Key Laboratory of Cardiovascular Remodeling and Function Research, Chinese Ministry of Education, Chinese National Health Commission and Chinese Academy of Medical Sciences, The State and Shandong Province Joint Key Laboratory of Translational Cardiovascular Medicine, department of Cardiology, Qilu Hospital of Shandong University, Jinan, Shandong China; 4 Department of Endocrinology and Metabolism, First People’s Hospital of Jinan City, Jinan, Shandong China

**Keywords:** Elderly, Type 2 diabetes, Glycemic variability, Arrhythmia

## Abstract

**Background:**

Little is known about whether the influence of glycemic variability on arrhythmia is related to age in type 2 diabetes mellitus (T2DM). Therefore, we aimed to compare the association between glycemic variability and arrhythmia in middle-aged and elderly T2DM patients.

**Methods:**

A total of 107 patients were divided into two groups: elderly diabetes mellitus group (EDM, *n* = 73) and middle-aged diabetes mellitus group (MDM, *n* = 34). The main clinical data, continuous glucose monitoring (CGM) and dynamic ECG reports were collected. The parameters including standard deviation of blood glucose (SDBG), largest amplitude of glycemic excursions (LAGE), mean amplitude of glycemic excursions (MAGE), absolute means of daily differences (MODD), time in range (TIR), time below range (TBR), time above range (TAR), coefficient of variation (CV) were tested for glycemic variability evaluation.

**Results:**

In terms of blood glucose fluctuations, MAGE (5.77 ± 2.16 mmol/L vs 4.63 ± 1.89 mmol/L, *P* = 0.026), SDBG (2.39 ± 1.00 mmol/L vs 2.00 ± 0.82 mmol/L, *P* = 0.048), LAGE (9.53 ± 3.37 mmol/L vs 7.84 ± 2.64 mmol/L, *P* = 0.011) was significantly higher in EDM group than those of MDM group. The incidences of atrial premature beat, couplets of atrial premature beat, atrial tachycardia and ventricular premature beat were significantly higher in EDM group compared with the MDM group (all *P* < 0.05). Among patients with hypoglycemia events, the incidences of atrial premature beat, couplets of atrial premature beat, atrial tachycardia and ventricular premature beat (all *P* < 0.05) were significantly higher in the EDM group than those in the MDM group. In EDM group, TIR was negatively correlated with atrial tachycardia in the MAGE1 layer and with atrial tachycardia and ventricular premature beat in the MAGE2 layer, TBR was significantly positively correlated with atrial tachycardia in the MAGE2 layer (all *P* < 0.05). In MDM group, TAR was positively correlated with ventricular premature beat and atrial tachycardia in the MAGE2 layer (all *P* < 0.05).

**Conclusions:**

The study demonstrated the elderly patients had greater glycemic variability and were more prone to arrhythmias. Therefore, active control of blood glucose fluctuation in elderly patients will help to reduce the risk of severe arrhythmia.

## Background

Diabetes mellitus (DM) is a major risk factor for the development of vascular complications. Recently, a series of studies showed that glycemic variability had more deleterious effects than sustained hyperglycemia in the pathogenesis of diabetic cardiovascular complications [1]. In patients of acute myocardial infarction with poor blood glucose control, notonlychronic hyperglycemia but also glycemic variability is associated with severity of coronary artery disease [2]. Another study demonstrated that glycemic variability predicted rapid progression of non-culprit lesions in patients with acute coronary syndrome [3]. Lu J et al. reported that glycemic variability was associated with the severity of diabetic retinopathy [4]. More recently, the ALLHAT study showed that greater visit-to-visit variability of fasting blood glucose was associated with increased risk of cardiovascular events and all-cause mortality [5]. Up to now, the mechanism by which glycemic variability aggravates the progression of cardiovascular disease is not fully understood, although several researches demonstrated that non-enzymatic glycation, oxidative stress, activation of inflammation and endothelial dysfunction might play a critical role [6, 7].

It is well known that patients with T2DM have a high risk of arrhythmias. Lately, the impact of glycemic variability on arrhythmia has attracted researchers’ attention. Stahn A et al. reported that severe episodes of hypoglycemia increased the risk of severe ventricular arrhythmias [8].In another study, silent hypoglycemia increased silent cardiac ischemia and the frequency of ventricular extrasystoles or nonsustained ventricular tachycardias [9]. Prolonged QT interval is a potential risk factor for malignant ventricular arrhythmias.Sertbas Y et al. found that increased glycemic variability was associated with prolonged QTc duration and QTc dispersion, suggesting that optimal glycemic control with glycemic variability should be considered as an additional goal point along with the traditional following parameters [10].

However, little is known about whether the influence of glycemic variability on arrhythmia is different between middle-aged and elderly T2DM patients with chronic cardiovascular disease. Therefore, in this study, middle-aged patients (MDM, aged 45–64 years) and elderly patients (EDM, aged 65 years or older) with diabetes combined with chronic cardiovascular disease were retrospectively included, and 72-h continuous dynamic blood glucose monitoring and 24-h continuous dynamic electrocardiogram monitoring were performed simultaneously in the hospitalized state to reveal the potential relationship.

## Method

### Subjects

This study groups enrolled 107 patients with known T2DM and chronic cardiovascular disease from February 2002 to September 2017 at Shandong University Qilu Hospital. This research was approved by the ethics committee of Qilu hospital, Shandong University and written informed consent was obtained before participation. Patients included in this study met all the following conditions: (1) Patients admitted to Qilu Hospital of Shandong University who met the 1998 World Health Organization (WHO) T2DM diagnostic criteria [11]; (2) Age ≥ 45 years; (3) clinically diagnosed chronic cardiovascular disease, including coronary heart disease, stroke, peripheral arterial disease or cerebrovascular disease [3]. Patients who met any of the followings were excluded from the study: (1) acute hyperbilirubinemia, ketoacidosis, lactic acidosis and other acute complications; (2) acute and chronic moderate and severe liver dysfunction, advanced tumor, anemia; (3) stress state caused by acute stroke, acute coronary syndrome, acute infection, trauma and perioperative period; (3) congenital heart disease, myocarditis; (4) permanent atrial fibrillation or atrioventricular block, baseline ECG ST segment depression greater than 1 mv; (5) pre-excitation syndrome; (6) installation of artificial cardiac pacing device; (7) taking antiarrhythmic drugs, such as propafenone, amiodarone, digitalis-like cardiac glycosides and other drugs that affect heart rate variability; (8) pregnant and lactating women; (9) mental illness such as epilepsy or neurosis; (10) electrolyte metabolism disorders; (11) thyroid dysfunction.

Clinical baseline index, such as age, gender, body weight, height, blood pressure and medical history, were obtained. The fasting blood samples were taken after an overnight fast of at least 12 h and sent to the biochemistry laboratory for estimation for glucose, hemoglobin A1c (HbA1C), lipid profiles, urea acid, and creatinine tests. Patients underwent 72-h continuous glucose monitoring (CGM, MiniMed MMT-7102 and MMMT-7102 W CGMS monitor of Medtronic, USA) and 24-h Holter (Philips Holter Plus/1810 Series) examination during hospitalization. The patients were in normal activity during the monitoring period, and the mealtime and exercise time were recorded. In the process of simultaneous CGM and dynamic ECG monitoring, the professional nurses guided the patients to make relevant clinical records. The dynamic blood glucose monitoring record was interpreted by an endocrinologist, and the Holter record was interpreted by a cardiologist.

Hypoglycemic event (HE) was Graded by peripheral random blood glucose: Grade 1 (3.1 mmol/L-3.9 mmol/L) and Grade 2 (< 3.1 mmol/L or suffering from severe symptoms requiring external intervention). HbA1c is difficult to reflect the information of blood glucose fluctuation and hypoglycemia [12]. CGM is the best method to evaluate blood glucose control in patients with high blood glucose fluctuation. Blood glucose variability is clearly recommended as one of the core indicators of CGM report [13], and the commonly used indicators of blood glucose fluctuation include the following four indicators [14]: standard deviations of blood glucose (SDBG, calculation method: standard deviation of measured values during CGM monitoring), large amplitude of glycemic excursion (LAGE, calculation method:the difference between the maximum and minimum blood glucose values during CGM monitoring), mean amplitude of glycemic excursion (MAGE, calculation method:after removing all blood glucose fluctuations of less than 1SDBG, the average value of blood glucose fluctuations was calculated according to the direction of the first effective fluctuation) and absolute means of daily differences (MODD, calculation method: The average absolute value of the subtraction between corresponding measured values over a continuous period of complete 48 h), time in range (TIR), time below range (TBR), time above range (TAR), coefficient of variation (CV) were calculated. The MAGE tertiles were layered as MAGE1 (1.24–4.37 mmol/l), MAGE2 (4.38–6.36 mmol/l) and MAGE3 (6.37–13.66 mmol/l) as previously reported [2].

The following arrhythmias were recorded: atrial premature beat (APB), Couplets of atrial premature beat (Couplets of A), atrial tachycardia (AT), ventricular premature beat (VPB), Couplets of ventricular premature beat (Couplets of V) and ventricular tachycardia (VT).

### Statistical analyses

Statistical analyses were performed using SPSS 19.0 (Chicago, IL, USA). Descriptive characteristics were presented as mean ± standard deviation (X ± SD). The Shapiro-Wilk normality test and the Levene variance homogeneity test are performed. For the variance in accordance with the normal distribution, the difference was compared using the independent sample t test. When the variance was not uniform, the difference between the two groups was compared by rank sum test. The χ2 test and Fisher exact test were used to determine differences in the proportion of categorical variables. *P* < 0.05 was considered as significantly different.

## Results

### Patient baseline characteristics

EDM group (*n* = 73): 43 patients received insulin, 13 patients used insulin secretagogues and 17 patients used other oral hypoglycemic agents. Forty-one patients with coronary heart disease, 15 patients with cerebrovascular disease, 10 patients combined with coronary heart disease or peripheral arterial disease, and 7 patients with peripheral arterial disease.

MDM group (*n* = 34): 19 cases used insulin, 6 cases treated with insulin secretagogue and 15 cases used other oral hypoglycemic agents. Eight cases with coronary heart disease, 2 cases with cerebrovascular disease, 8 patients combined with coronary heart disease and peripheral arterial disease, and 16 cases with peripheral arterial disease.

### Comparison of the clinical characteristics between patients in EDM and MDM groups

The BMI (25.68 ± 3.23 kg/m^2^ vs 27.29 ± 3.50 kg/m^2^, *P* = 0.021) and DBP levels (68.25 ± 9.21 mmHg νs 77.47 ± 14.41 mmHg, *P* = 0.001) of the EDM group were significantly lower, while the prevalence of hypertension (HP) (79.5% vs 52.9%, *P* = 0.005) were significantly higher than those of the MDM group. There was no significant difference in fasting blood glucose, hemoglobin A1c, uric acid, creatinine and blood lipid levels between the two groups (Table 1).

**Table 1:** Comparison of general clinical data of EDM and MDM patients

Variables	EDM	MDM	*P*
n	73	34	
Male [n,(%)]	56 (76.7)	22 (64.7)	0.536
Age(yrs)	81.08 ± 5.36	56.68 ± 5.24	< 0.001
BMI (kg/m)	25.68 ± 3.23	27.29 ± 3.50	0.021
SBP (mmHg)	142.03 ± 18.20	135.26 ± 23.12	0.139
DBP (mmHg)	68.25 ± 9.21	77.47 ± 14.41	0.001
Anti-diabetes drugs			
sulfonylurea [n, (%)]	13 (17.8)	6 (17.6)	0.507
Insulin [n, (%)]	43 (58.9)	19 (55.9)	0.528
Others [n, (%)]	17 (35.6)	9 (26.5)	0.223
Metformin	10	7	0.3640
α-glucosidase inhibitor	5	1	0.6621
DPP 4 inhibitors	1	1	0.5366
TZD	1	0	1.0000
Other drugs			
CCB	12	3	0.3787
ACEI	11	3	0.5410
ARB	17	5	0.3064
CCB + ACEI	0	2	0.0989
CCB + ARB	18	5	0.2433
ASP	28	12	0.7605
Statins/Brates	17	7	0.7553
HP [n, (%)]	58 (79.5)	18 (52.9)	0.005
FBG (mmol/L)	7.21 ± 3.30	7.33 ± 3.05	0.649
HbA_l_C (%)	7.47 ± 1.70	7.69 ± 2.16	0.456
TC (mmol/L)	4.38 ± 1.16	4.69 ± 1.05	0.375
TG (mmol/L)	1.45 ± 0.94	2.01 ± 1.07	0.012
HDL-C (mmol/L)	1.23 ± 0.28	1.21 ± 0.41	0.830
LDL-C (mmol/L)	2.53 ± 0.89	2.57 ± 0.79	0.284
UA (umol/L)	335.16 ± 99.02	321.13 ± 91.65	0.501
Cr (umol/L)	80.07 ± 17.71	74.29 ± 25.65	0.655

The difference was compared using the independent sample *t* test or χ2 test

*BMI* body mass index, *SBP* systolic blood pressure, *DBP* diastolic blood pressure, *TZD* thiazolidinedione, *CCB* Calcium Channel Blocker, *ACEI* angiotensin-converting enzyme inhibitor, *ARB* angiotensin receptor blocker, *HP* hypertension, *FBG* fast blood glucose, *HbA1C* hemoglobin A1c, *TC* total cholesterol, *TG* triglyceride, *HDL-C* high density lipoprotein cholesterol, *LDL-C* low density lipoprotein cholesterol, *UA* uric acid, *Cr* Creatinine

### Comparison of hypoglycemia between EDM and MDM groups

The incidences of grade 1 hypoglycemia (55.9% vs 64.7%, *P* = 0.403) and grade 2 hypoglycemia (15.1% vs 20.6%, *P* = 0.477) in EDM group were lower than those of the MDM group, but the differences were not statistically significant. For the occurrence time of hypoglycemia, 20 case-times occurred during the day and 23 case-times occurred at night in the EDM group, and in the MDM group, 13 case-times occurred during the day and 11 case-times occurred at night. (6 am-10 pm during the day; 10 pm-6 am at night).

### Comparison of blood glucose fluctuation indicators between EDM and MDM groups

The SDBG (2.39 ± 1.00 mmol/L vs 2.00 ± 0.82 mmol/L, *P* = 0.048), LAGE (9.53 ± 3.37 mmol/L vs 7.84 ± 2.64 mmol/L, *P* = 0.011) and MAGE (5.77 ± 2.16 mmol/L vs 4.63 ± 1.89 mmol/L, *P* = 0.026) in EDM group were significantly higher than those of the MDM group. These results suggested that patients of EDM have greater blood glucose fluctuations (Table 2).

**Table 2:** Comparison of the blood glucose variability index between the groups of EDM and MDM (mean ± SD)

Variables	EDM	MDM	*P*
n	73	31	
SDBG (mmol/L)	2.39 ± 1.00	2.00 ± 0.82	0.048
LAGE (mmol/L)	9.53 ± 3.37	7.84 ± 2.64	0.011
MAGE (mmol/L)	5.77 ± 2.16	4.63 ± 1.89	0.026
MODD (mmol/L)	2.13 ± 1.09	1.72 ± 1.03	0.063
TIR (%)	63.92 ± 28.32	61.35 ± 26.78	0.626
TAR (%)	37.60 ± 28.52	38.03 ± 26.99	0.531
TBR (%)	1.16 ± 2.75	0.53 ± 1.40	0.489
CV (%)	24.86 ± 8.1	25.74 ± 8.4	0.424

The difference was compared using the independent sample t test

*SDBG* Standard Deviations of Blood Glucose, *LAGE* Large Amplitude of Glycemic Excursion, *MAGE* Mean Amplitude of Glycemic Excursion, *MODD* Absolute Means of Daily Differences, *TIR* time in range, *TBR* time below range, *TAR* time above range, *CV* coefficient of variation

### Comparison of heart rate and arrhythmia between EDM and MDM groups

The maximum heart rate (100.26 ± 13.73 vs 112.27 ± 20.75, *P* = 0.017) and average heart rate (67.93 ± 9.82 vs 73.22 ± 9.03, *P* = 0.026) in the EDM group were significantly lower than those in the MDM group. There was no significant difference of the minimum heart rate between the two groups. The incidences of atrial premature beat, couplets of atrial premature beat, atrial tachycardia, ventricular premature beat and couples of ventricular premature beat in the EDM group were significantly higher than those in the MDM group (all *P* < 0.05). The mean QTc interval (0.47 ± 0.01 vs 0.45 ± 0.00, *P* = 0.029) in the EDM group was significantly longer than that of the MDM group (Table 3).

**Table 3:** Comparison of arrhythmia indicators between the groups of EDM and MDM

	n	Maximal heart rate (mean ± SD)	Minimal heart rate (mean ± SD)	Mean heart rate (mean ± SD)	APB [M(Q1, Q3)]	Couplets of A [M(Q1, Q3)]	AT [M(Q1, Q3)]	VPB [M(Q1, Q3)]	Couplets of V [M(Q1, Q3)]	VT [M(Q1, Q3)]	Mean QTc (s) (mean ± SD)
EDM	73	100.26 ± 13.73	51.84 ± 9.99	67.93 ± 9.82	135 (35,382)	2 (1,8)	1 (0,3)	25 (3,303)	0 (0,0)	0 (0,0)	0.47 ± 0.01
MDM	34	112.27 ± 20.75	56.82 ± 17.84	73.22 ± 9.03	7 (2,18)	0 (0,0)	0 (0,0)	1 (0,3)	0 (0,0)	0 (0,0)	0.45 ± 0.00
*P*		0.017	0.222	0.026	<0.001	<0.001	<0.001	<0.001	0.034	0.122	0.029

The difference was compared by independent sample *t* test or rank sum test

*APB* atrial premature beats, *Couplets of A* Couplets of atrial premature beats, *AT* atrial tachycardia, *VPB* ventricular premature beats, *Couplets of V* Couplets of ventricular premature beats, *VT* ventricular tachycardia

### Comparison of arrhythmias between EDM group and MDM group under different MAGE layers

In patients of MAGE1 (1.24–4.37 mmol/L), the number of atrial premature beat (*P* = 0.012) was significantly higher in the EDM group. In patients of MAGE2 (4.38–6.36 mmol/L), atrial premature beat (*P* < 0.001), couples of atrial premature beat (*P* < 0.001), atrial tachycardia (*P* = 0.001), and ventricular premature beat (*P* < 0.001) occurred significantly more frequent in the EDM group. The QTc interval (0.48 ± 0.01 s vs 0.45 ± 0.01 s, *P* = 0.047) was significantly longer in the EMD group. In patients of MAGE3 (6.37–13.66 mmol/L), we also found significant higher incidences of atrial premature beat (*P* < 0.001), couples of atrial premature beat (*P* < 0.001), atrial tachycardia (*P* < 0.001), and ventricular premature beat (*P* = 0.013) in EDM group (Table 4).

**Table 4:** Difference in arrhythmia according to MAGE and the tertiles MAGE between the groups of EDM and MDM

Variables		EDM	MDM	*P*
n		28	8	
MAGE1	APB [M(Q1, Q3)]	129 (44.5228)	18 (4,51)	0.012
	Couplets of A [M(Q1, Q3)]	2.5 (1,7)	2 (0,4.5)	0.256
	AT [M(Q1, Q3)]	1 (0,4)	0.5 (0,1.5)	0.127
	VPB [M(Q1, Q3)]	21 (2237.5)	0 (0,49)	0.083
	Couplets of V[M(Q1, Q3)]	0 (0,0)	0 (0,0)	0.279
	VT [M(Q1, Q3)]	0 (0,0)	0 (0,0)	0.472
	Mean QTc (s)(mean ± SD)	0.45 ± 0.01	0.46 ± 0.01	0.362
	n	24	12	
MAGE2	APB [M(Q1, Q3)]	108 (23.5450)	7 (2,15.5))	<0.001
	Couplets of A [M(Q1, Q3)]	2 (0,5)	0 (0,0)	<0.001
	AT [M(Q1, Q3)]	1 (0,3)	0 (0,0)	0.001
	VPB [M(Q1, Q3)]	97.5 (4923)	1.5 (0,3)	<0.001
	Couplets of V[M(Q1, Q3)]	0 (0,0)	0 (0,0)	0.221
	VT [M(Q1, Q3)]	0 (0,0)	0 (0,0)	0.331
	Mean QTc (s)(mean ± SD)	0.48 ± 0.01	0.45 ± 0.01	0.047
	n	21	14	
MAGE3	APB [M(Q1, Q3)]	210 (46,750)	5.5 (4,9)	<0.001
	Couplets of A[M(Q1, Q3)]	4 (1,28)	0 (0,0)	<0.001
	AT [M(Q1, Q3)]	2 (1,7)	0 (0,0)	<0.001
	VPB [M(Q1, Q3)]	7 (2196)	0.5 (0,4)	0.013
	Couplets of V [M(Q1, Q3)]	0 (0,0)	0 (0,0)	0.259
	VT [M(Q1, Q3)]	0 (0,0)	0 (0,0)	0.448
	Mean QTc (s)(mean ± SD)	0.47 ± 0.01	0.45 ± 0.01	0.234

The difference was compared by rank sum test

*APB* atrial premature beats, *Couplets of A* Couplets of atrial premature beats, *AT* atrial tachycardia, *VPB* ventricular premature beats, *Couplets of V* Couplets of ventricular premature beats, *VT* ventricular tachycardia

### Comparison of arrhythmias in patients with hypoglycemic events between EDM and MDM groups

The maximum heart rate (99.52 ± 12.47 beat/min vs 111.83 ± 18.08 beat/min, *P* = 0.006) and mean heart rate (67.60 ± 9.28 beat/min vs 74.26 ± 8.93 beat/min, *P* = 0.007) in EDM patients were significantly lower than those of MDM patients, while the minimum heart rate was not significantly different. The incidences of atrial premature beat (*P* < 0.001), couples of atrial premature beat (*P* < 0.001), atrial tachycardia (*P* < 0.001) and ventricular premature beat (*P* = 0.002) in patients with EDM were significantly higher than those in the MDM group (Table 5).

**Table 5:** Comparison of arrhythmias in patients with HE in groups of EDM and MDM

HE	n	Maximal heart rate (mean ± SD)	Minimal heart rate (mean ± SD)	Mean heart rate (mean ± SD)	APB [M(Q1, Q3)]	Couplets of A [M(Q1, Q3)]	AT [M(Q1, Q3)]	VPB [M(Q1, Q3)]	Couplets of V [M(Q1, Q3)]	VT [M(Q1, Q3)]	Mean QTc (s) (mean ± SD)
EDM	42/73	99.52 ± 12.47	53.14 ± 9.41	67.60 ± 9.28	120 (34,379)	2 (1,6)	1 (0,3)	14.5 (2,278)	0 (0,0)	0 (0,0)	0.45 ± 0.01
MDM	23/34	111.83 ± 18.08	58.09 ± 17.19	74.26 ± 8.93	7 (2,22)	0 (0,2)	0 (0,0)	1 (0,14))	0 (0,0)	0 (0,0)	0.46 ± 0.01
*P*		0.006	0.211	0.007	<0.001	<0.001	<0.001	0.002	0.199	0.302	0.135

The difference was compared by independent sample *t* test or rank sum test

*HE* hypoglycemic event, *APB* atrial premature beats, *Couplets of A* Couplets of atrial premature beats, *AT* atrial tachycardia, *VPB* ventricular premature beats, *Couplets of V* Couplets of ventricular premature beats, *VT* ventricular tachycardia

### Correlation analysis of TIR, TAR, TBR CV and arrhythmia

In the MAGE1 layer of EDM, atrial tachycardia was negatively correlated with TIR (*P* = 0.042). In the MAGE2 layer of EDM, TIR was significantly negatively correlated with atrial tachycardia (*P* = 0.047) and ventricular premature beat (*P* = 0.037), TBR was significantly positively correlated with atrial tachycardia (*P* = 0.041), while in the MAGE2 layer of MDM, TAR was significantly positively correlated with ventricular premature beat (*P* = 0.028) and atrial tachycardia (*P* = 0.046) (Tables 6 and 7).

**Table 6:** Correlation analysis of TIR, TAR, TBR, CV and arrhythmia between the groups of EDM and MDM

Variables	EDM		MDM		EDM		MDM		EDM		MDM		EDM		MDM	
	TIR		TIR		TAR		TAR		TBR		TBR		CV		CV	
	*r*	*P*	*r*	*P*	*r*	*P*	*r*	*P*	*r*	*P*	*r*	*P*	*r*	*P*	*r*	*P*
APB	0.190	0.109	− 0.129	0.466	− 0.163	0.171	0.134	0.451	0.025	0.834	−0.242	0.168	0.043	0.717	−0.239	0.172
Couplets of A	0.014	0.906	−0.190	0.279	0.017	0.889	0.194	0.272	−0.041	0.732	−0.277	0.112	0.020	0.866	0.149	0.402
AT	0.121	0.311	−0.141	0.428	−0.102	0.393	0.158	0.372	− 0.056	0.642	−0.256	0.144	−0.022	0.949	0.084	0.635
VPB	0.037	0.755	−0.167	0.344	−0.035	0.767	0.159	0.369	0.052	0.662	0.108	0.541	− 0.052	0.663	0.044	0.805
Couplets of V	−0.174	0.144	–	–	0.170	0.153	–	–	0.089	0.453	–	–	0.067	0.576	–	–
VT	0.041	0.733	–	–	−0.033	0.783	–	–	−0.045	0.709	–	–	−0.059	0.614	–	–

*TIR* Time In Range, *TBR* Time Below Range, *TAR* Time Above Range, *CV* coefficient of variation, *APB* atrial premature beats, *Couplets of A* Couplets of atrial premature beats, *AT* atrial tachycardia, *VPB* ventricular premature beats, *Couplets of V* Couplets of ventricular premature beats, *VT* ventricular tachycardia

**Table 7:** Correlation analysis of TIR, TAR, TBR and arrhythmia in tertiles MAGE in MDM and EDM

Variables		EDM		MDM		EDM		MDM		EDM		MDM		EDM	MDM
		TIR		TIR		TAR		TAR		TBR		TBR		CV	CV
		*r*	*P*	*r*	*P*	*r*	*P*	*r*	*P*	*r*	*P*	*r*	*P*	*r*	*P*
MAGE1	APB	0.122	0.571	− 0.066	0.846	− 0.049	0.819	0.066	0.846	−0.159	0.456	− 0.547	0.080	− 0.548	0.081
	Couplets of A	0.010	0.961	−0.222	0.511	0.057	0.792	0.222	0.511	−0.356	0.088	−0.565	0.070	−0.565	0.070
	AT	−0.417	0.042	−0.297	0.375	−0.382	0.065	0.297	0.375	−0.240	0.258	−0.108	0.752	−0.108	0.752
	VPB	0.149	0.485	−0.443	0.172	−0.149	0.486	0.443	0.172	− 0.036	0.869	0.253	0.453	0.252	0.453
	Couplets of V	−0.136	0.524	–	–	0.192	0.368	–	–	0.078	0.717	–	–	–	–
	VT	0.361	0.084	–	–	−0.316	0.132	–	–	− 0.153	0.474	–	–	–	–
MAGE2	APB	0.287	0.174	0.208	0.515	−0.267	0.208	−0.190	0.553	0.169	0.428	0	1	−0.176	0.584
	Couplets of A	0.068	0.753	0.480	0.114	−0.064	0.767	−0.480	0.113	0.135	0.528	−0.172	0.593	0.218	0.495
	AT	−0.583	0.047	0.061	0.776	−0.017	0.937	0.583	0.046	0.594	0.041	−0.255	0.424	0.388	0.211
	VPB	−0.605	0.037	−0.140	0.513	0.159	0.457	0.630	0.028	−0.245	0.248	0.056	0.863	0.171	0.595
	Couplets of V	−0.256	0.226	–	–	0.256	0.226	–	–	−0.106	0.621	–	–	–	–
	VT	–	–	–	–	–	–	–	–	–	–	–	–	–	–
MAGE3	APB	0.172	0.421	−0.266	0.429	−0.189	0.374	0.266	0.429	0.066	0.758	−0.402	0.220	−0.485	0.130
	Couplets of A	0.029	0.890	−0.296	0.376	−0.038	0.858	0.296	0.376	0.046	0.830	−0.365	0.269	−0.104	0.760
	AT	0.076	0.725	−0.277	0.409	−0.104	0.623	0.276	0.410	0.212	0.317	−0.367	0.267	−0.087	0.799
	VPB	0.032	0.881	0.063	0.854	−0.083	0.697	−0.063	0.854	0.275	0.192	0.230	0.496	−0.313	0.347
	Couplets of V	− 0188	0.378	–	–	0.144	0.500	–	–	0.159	0.455	–	–	–	–
	VT	−0.144	0.503	–	–	0.126	0.557	–	–	−0.063	0.771	–	–	–	–

The MAGE tertiles were layered as MAGE1 (1.24–4.37 mmol/l), MAGE2 (4.38–6.36 mmol/l) and MAGE3 (6.37–13.66 mmol/l)

*TIR* Time In Range, *TBR* Time Below Range, *TAR* Time Above Range, *CV* coefficient of variation, *APB* atrial premature beats, *Couplets of A* Couplets of atrial premature beats, *AT* atrial tachycardia, *VPB* ventricular premature beats, *Couplets of V* Couplets of ventricular premature beats, *VT* ventricular tachycardia

## Discussion

Controlling blood glucose fluctuations in diabetic patients has gradually become an emerging therapeutic target for improving diabetes metabolism and preventing related complications [15]. Large cohort studies have shown that blood glucose fluctuations are not only an important predictor of mortality in diabetic patients but also in non-diabetic patients [16]. MAGE is the “gold standard” which can best reflect the blood glucose fluctuation [17]. However, up to now, there are few reports comparing the effects of glycemic variability on arrhythmias between the middle-aged and the elderly patients. Our current study found that the levels of SDBG, LAGE and MAGE in EDM group were statistically higher than those in MDM group, indicating the greater blood glucose fluctuations in EDM patients. Accordingly, the incidences of supraventricular and ventricular arrhythmia were higher in EDM groups. Thus, it may be more meaningful to monitor the blood glucose fluctuations and act corresponding treatment to prevent severe arrhythmia in elder T2DM patients.

ADVANCE study found that T2DM patients with severe hypoglycemia had a threefold increased risk of major cardiovascular events and a twofold increased risk of microvascular complications [18]. History of episodes of severe hypoglycemia was associated with increased incidence of new atrial fibrillation and all-cause mortality [19]. In patients with T2DM complicated with cardiovascular disease, the incidence of bradycardia, atrial premature beat and ventricular premature beat was high, especially when spontaneous hypoglycemia occurs in patients during sleep at night [20]. Due to the course of disease, age and other reasons, the decline of β cell function in EDM patients is more obvious than that in young and middle-aged T2DM patients, which may lead to the greater amplitude of blood glucose fluctuation [17]. Our research shows that although the incidence of hypoglycemia was not obvious different in patients between EDM and MDM groups, the incidences of atrial premature beat, couples of atrial premature beat, atrial tachycardia and ventricular premature beat were significant higher in EDM patients, suggesting the elderly patients were more prone to supraventricular and ventricular arrhythmia.

Prolonged cardiac repolarization causes fatal cardiac arrhythmias. The prolongation of QTc is a strong risk factor for severe ventricular arrhythmia and sudden death. Studies have shown that experimental hypoglycemia caused acquired long QTc syndrome [21], probably through sympathoadrenal stimulation and catecholamine-mediated hypokalemia. Up to now, few studies showed the association between glycemic variability and QT interval and dispersion. Ninkovic et al. mentioned about the GV as a risk factor of prolonged QT interval and QT dispersion [22]. Another study showed that in patients with T2DM, increased glycemic variability is associated with prolonged QTc duration and QTc dispersion [10]. Our present study demonstrated that the average QTc interval of EDM was significantly longer than that of MDM patients, suggesting that the elderly subjects were prone to arrhythmia than the middle-aged subjects.

Previous studies found that the rapid fluctuation of average blood glucose fluctuation (MAGE> 5 mmol/L) increased the vulnerability of the electrical stability of the heart, leading to higher incidence of arrhythmia, such as atrial fibrillation [23, 24]. In our study, the incidences of atrial premature beat, couples of atrial premature beat, atrial tachycardia and ventricular premature beat of EDM patients in MAGE2 and MAGE3 groups were significantly higher than those of MDM patients. Our study also analyzed the incidence of arrhythmia between EDM and MDM patients with HE. The incidences of atrial premature beat, couples of atrial premature beat, atrial tachycardia and ventricular premature beat in EDM patients were significantly higher than those in MDM patients. In addition, our current study found that TIR was significantly negatively correlated with atrial tachycardia and ventricular premature beat, TBR was significantly positively correlated with atrial tachycardia in the elderly subjects. These results suggested that the elderly patients with moderate or high blood glucose fluctuations are more likely to cause arrhythmias.

The limitation of this study should be also considered. The dynamic ECG only collects 24-h ECG data, thus dynamic blood glucose also only analyses the corresponding 24-h data. In future studies, we will continue to synchronize dynamic ECG and dynamic blood glucose monitoring for 72 h in order to more accurately reflect the relationship between blood glucose fluctuation and arrhythmia, and further explore the correlation between abnormal blood glucose fluctuation and complications such as macrovascular, microvascular and nervous system in elderly patients with T2DM.

## Conclusions

The elderly patients with cardiovascular disease had greater fluctuation of blood glucose and the incidences of arrhythmia. Therefore, active control of blood glucose fluctuation in elderly patients will help to reduce the risk of severe arrhythmia.

## Data Availability

The datasets used and/or analyzed during the present study are available from the corresponding author on reasonable request.

## Abbreviations

CGM: Continuous blood glucose monitoring
ECG: Electrocardiogram
T2DM: Type 2 diabetes
EDM: Elderly diabetes mellitus group
MDM: Middle-aged diabetes mellitus
SDBG: Standard deviation
LAGE: Largest amplitude of glycemic excursions
MAGE: Mean amplitude of glycemic excursions
MODD: Absolute means of daily differences
TIR: Time in range
TBR: Time below range
TAR: Time above range
CV: Coefficient of variation
WHO: World Health Organization
HE: Hypoglycemic events
APB: Atrial premature beat
Couplets of A: Couplets of atrial premature beat
AT: Atrial tachycardia
VPB: Ventricular premature beat
Couplets of V: Couplets of ventricular premature beat
VT: Ventricular tachycardia
BMI: Body mass index
SBP: Systolic blood pressure
DBP: Diastolic blood pressure
HP: Hypertension
FBG: Fast blood glucose
HbA1C: Hemoglobin A1c
TC: Total cholesterol
TG: Triglyceride
HDL-C: High density lipoprotein cholesterol
LDL-C: Low density lipoprotein cholesterol
UA: Uric acid
Cr: Creatinine
